# Utility of Antidepressant Randomized Controlled Trials for RegularTreatment

**DOI:** 10.1055/a-2838-5092

**Published:** 2026-04-21

**Authors:** Anton J. M. Loonen

**Affiliations:** 1 Pharmacotherapy, -Epidemiology & -Economics 3647 University of GroningenGroningenNetherlands

In the Western world, responsibilities regarding pharmacotherapy are divided: the government ensures that only effective and safe medicines are available, while healthcare professionals—doctors and other prescribers—are responsible for ensuring that these medicines are used correctly. In the era of evidence-based medicine (EBM), healthcare providers are expected to follow diagnosis and treatment guidelines provided by professional associations like the American Psychiatric Association or institutes such as the National Institute for Health and Care Excellence in the United Kingdom. With regard to the pharmacotherapy of depressive disorders, these guidelines are based on evidence from randomised controlled trials (RCTs) of antidepressants, the same trials that are used to grant drugs marketing authorization. In this commentary, the author presents arguments for revising this approach.

In medicine, the RCT is widely considered the most reliable method. For this reason, regulatory authorities require two short-term pivotal RCTs to demonstrate an antidepressant's efficacy. 1​ Additionally, studies are required to assess the drug's ability to prevent relapse with continued treatment and recurrence during maintenance therapy. However, this commentary does not address those two types of research.

What is the typical design of an RCT intended to demonstrate the therapeutic effect of a depression treatment?. 1​ Generally, a randomized, double-blind, placebo-controlled, parallel group design is used. Participants are selected based on the severity of depression and their diagnostic category. These categories were established because previous research showed that certain disorders responded better to imipramine— the prototype antidepressant—than to a placebo. 2​ ​3​ The severity of depression is estimated using rating scales, such as the 17-item Hamilton Depression Rating Scale. The response is based on the decrease in score after approximately 6 weeks of treatment. Remission is rarely used as an outcome measure; instead, the response is usually defined as a 50% or greater reduction in score. 1​ Since participation in these trials is strictly voluntary and participants are uncertain about the nature of their medication, and other reasons, many drop out prematurely.

European registration authorities require that patients in studies be diagnosed with major depressive disorder (MDD) based on widely accepted diagnostic criteria. 1​ These are often the criteria from the Diagnostic and Statistical Manual of Mental Disorders (DSM). 4​ The definition of these criteria has significantly broadened over time. In the third edition of the DSM, published in 1980, a separate category called “MDD with melancholia” was created. The DSM-III text indicates that this is a subtype that responds preferentially to “somatic” therapies; at that time, tricyclic antidepressants or electroconvulsive therapy. 5​ ​6​ This category, derived from the Research Diagnostic Criteria, 7​ intended to select individuals whose depression would respond to these somatic therapies but not to a placebo. This idea of the “Kraepelinian school of thought” was rooted in the work of psychiatrist Emil Kraepelin (1856–1926), who proposed that clinical syndromes, including depression, 8​ could be classified into distinct categories in which a predictable relationship exists among the symptoms, course, aetiology and pathogenesis of the mental illness. 9​ ​10​ The depression was referred to as “Melancholie” as part of “Manisch-depressives Irresein”. 10​ While these Kraepelinian ideas were contested by, among others, followers of Adolf Meyer (1866–1955), 10​ it is plausible that the DSM-III “MDD with melancholia” category only loosely overlaps with the “Melancholie” studied and/or referred to by Kraepelin in the early 20 ^th^ century. Kraepelin's patients typically came from psychiatric (or later academic) hospitals, meaning most patients with 1980's DSM-III “MDD with melancholia” would not have belonged to his caseload in those days. This is even truer for contemporary MDD as defined in the DSM-5 TR from 2022. 11​ The world and humanity have changed too much and this undoubtedly also influences the nature of current psychopathology.

The concept of depression is deeply rooted in Western culture, with descriptions dating back to ancient times. 12​ Gregory the Great (Pope 590–604 AD) dismissed something resembling clinical depression, known as “tristitia,” as a mortal sin, alongside the Hippocratic-Galenic concept of melancholia, which had a more physical basis. 13​ ​14​ It is likely that many individuals in the early middle ages who were accused of committing this sin would be diagnosed with at least dysthymia and in my opinion also MDD in 2022. 13​ Because this belief persisted in the second half of the 20 ^th^ century, a significant proportion of current MDD cases involves conditions that would not have been treated with medical interventions, such as antidepressants, but rather with clerical or psychological support. Although in this earlier situation some people with depression may have been under-treated, the current abandonment of restraint with antidepressants has undoubtedly led to unnecessary medicalisation. This history challenges the idea of MDD as a reliable indicator for prescribing antidepressants, leading to a large placebo response. While I do not dispute that RCTs showing a difference between verum and placebo are valid, I believe that the size of this difference holds little significance. It also means that no definitive statement can be made about which treatment strategy is most effective. 15​

Shortly after the discovery of imipramine's antidepressant effects, the pharmacological target of antidepressant drugs became the starting point for understanding the pathophysiological mechanisms underlying depressive disorder. This led to the development of the monoamine hypothesis for endogenous depression, which dominated biological psychiatry for decades. 16​ Since then, at least four other hypotheses have emerged, 16​ each suggesting that different biological interventions with varying points of action may also have antidepressant effects. A similar observation led Hans H. Stassen and colleagues at the Psychiatric University Hospital in Zurich, Switzerland, to conclude that “affectively ill patients are likely to possess a common, biological, ‘resilience'-like component that largely controls recovery from depression”. 17​ They based this idea on RCTs comparing seven different antidepressants with placebo in a total of 2,848 patients with MDD (according to DSM criteria). They found no significant difference in the response patterns between users of verum and placebo; the mean±standard deviation time to the onset of 20% improvement was 13±1 days, and to 50% response was 19±1 days. This suggests that antidepressants may activate the same biological process that drives recovery when using a placebo.

I find the idea compelling that all kinds of treatments (biological, psychotherapeutic, and pastoral religious) could lead to improvement by activating a universal, natural ‘resilience' mechanism in patients with MDD. Depressive reactions to disappointments, losses, and/or setbacks are certainly not uncommon in everyday life. Almost always, individuals have behavioural tools at their disposal to recover quickly from these depressive symptoms. When this mood decline persists, the activation of a natural resilience mechanism could be the final common pathway for recovery across different treatment types.

If one accepts this model, which suggests that antidepressant treatment promotes a natural, biological resilience mechanism, another issue arises when interpreting standard RCTs on antidepressant efficacy. Since, in these RCTs, the spontaneous recovery as part of the placebo response could make the difference between the placebo and the active treatment too small, unknowingly efforts are made to minimize spontaneous recovery as part of the placebo effect. When significant differences are observed between products, they may reflect the degree to which the placebo effect was minimized in the studied population. The most accurate approach would be to focus on the magnitude of the placebo response, but unfortunately, such head-to-head comparisons do not always include a placebo group.

As mentioned earlier, we are in the era of EBM. One of EBM's main objectives is: “the conscientious, explicit, and judicious use of the best available evidence in the decision-making process for the care of individual patients”. 18​ The highest level of evidence is typically derived from a systematic review of multiple RCTs, which is now complemented by network meta-analyses. While more complex, these analyses, if done correctly, are considered more meaningful 19​ ​20​ However, a major weakness is that the aggregated RCTs. are all broadly designed according to the same template as the RCTs for registration purposes. For pharmacotherapy, the same studies serve both purposes. As noted earlier, these RCTs have two interrelated limitations that make it difficult to accurately determine the extent of the therapeutic effect: the heterogeneity that remains possible within MDD criteria and the extent to which the natural biological resilience mechanism can manifest. Many of these limitations carry over into meta-analyses. In my view, these meta-analyses do not provide the best available evidence for determining treatment strategies.

How, then, can depression treatment guidelines be developed without over-relying on these standard RCTs? I believe that the experiences gained in daily practice should be more fully utilized. Pragmatic trials like the legendary Sequenced Treatment Alternatives to Relieve Depression study 21​ are already moving in this direction, but this approach could be enhanced by standardising diagnosis, treatment and outcome measures within a “fixed group of primary and secondary care practitioners,” focusing on drug treatment-naive patients. In patients with prior treatment histories, previous responses to pharmacotherapy should be considered. A significant shift from traditional RCTs could be achieved by focusing primarily on individuals who do not achieve full remission within a reasonable time – although the timeline for this will vary depending on the type of non-response. 21​ Of course, any potential causes for non-response, such as poor adherence, must first be investigated. If no obvious cause is identified, the group should be further researched to determine the exact nature of the non-response, mirroring the approach used in daily practice. Diagnostic tools and treatment decisions should ideally reflect their utility in real-world practice, where most experience and confidence lie. When new treatments, based on RCTs, are expected to outperform older ones, they can replace previous treatments in the pragmatic trial, using past experience as a historical control.

## Statement

During the preparation of this work, the author used DeepL Translate ( https://www.deepl.com/nl/translator) in order to increase the variability of English language formulations. The language of the final text was edited by Elsevier Author Services (https://scientific-publishing.webshop.elsevier.com/). After using this tool and service, the author reviewed and edited the content as needed and takes full responsibility for the content of the publication.

## Note

Letters to the editor do not necessarily represent the opinion of the editor or publisher. The editor and publisher reserve the right to not publish letters to the editor, or to publish them abbreviated or in extracts.
